# Mechanism of microRNA‐22 in regulating neuroinflammation in Alzheimer’s disease

**DOI:** 10.1002/brb3.1627

**Published:** 2020-04-19

**Authors:** Chenyang Han, Li Guo, Yi Yang, Qiaobing Guan, Heping Shen, Yongjia Sheng, Qingcai Jiao

**Affiliations:** ^1^ State Key Laboratory of Pharmaceutical Biotechnology School of Life Science Nanjing University Nanjing China; ^2^ Department of Pharmacy The Second Affiliated Hospital of Jiaxing University Jiaxing China; ^3^ Department of Center Laboratory The Second Affiliated Hospital of Jiaxing University Jiaxing China; ^4^ Department of Neurology The Second Affiliated Hospital of Jiaxing University Jiaxing China

**Keywords:** Alzheimer's disease, inflammatory factor, microglia, microRNA‐22, pyroptosis

## Abstract

**Background:**

Study on the expression of miRNA‐22 in serum of Alzheimer's disease (AD) patients and the mechanism of neuroinflammation regulation.

**Methods:**

ELISA assay was used to detect the serum level of inflammatory factors, including interleukin‐1β (IL‐1β), interleukin‐18 (IL‐18), and tumor necrosis factor‐α in AD patients. TargetScan database and luciferase reporter gene assay indicated that gasdermin D (GSDMD) was the target gene of miRNA‐22. miRNA‐22 mimic was transfected into microglia, followed by administration of LPS and Nigericin to induce pyroptosis.

**Results:**

In this study, we found that the expression level of miRNA‐22 in peripheral blood was lower in AD patients than that in healthy population. The expression of inflammatory factors was higher in AD patients than that in healthy people, which was negatively correlated with miRNA‐22. miRNA‐22 mimic could significantly inhibit pyroptosis, the expression of GSDMD and p30‐GSDMD was down‐regulated, the release of inflammatory factor was decreased, and the expression of NLRP3 inflammasome was down‐regulated as feedback. In the APP/PS1 double transgenic mouse model, the injection of miRNA‐22 mimic significantly improved the memory ability and behavior of mice. In addition, the expression of the vital protein of pyroptosis in mouse brain tissue, including GSDMD and p30‐GSDMD, was down‐regulated, and the expression of inflammatory factors was also decreased.

**Conclusion:**

miRNA‐22 was negatively correlated with the expression of inflammatory factors in AD patients, and miRNA‐22 could inhibit the release of inflammatory cytokines by regulating the inflammatory pyroptosis of glial cells via targeting GSDMD, thereby improving cognitive ability in AD mice. miRNA‐22 and pyroptosis are potential novel therapeutic targets in the treatment of AD.

## Graphical abstract

miRNA‐22 was negatively correlated with the expression of inflammatory factors in Alzheimer's disease (AD) patients, and miRNA‐22 could inhibit the release of inflammatory cytokines by regulating the inflammatory pyroptosis of glial cells via targeting GSDMD, thereby improving cognitive ability in AD mice. miRNA‐22 and pyroptosis are potential novel therapeutic targets in the treatment of AD.

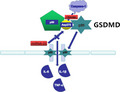

## 1 INTRODUCTION

Alzheimer's disease (AD) is a chronic neurodegenerative disease, clinically characterized by memory impairment, cognitive impairment, visual spatial ability, and abstract thinking damage (Guerriero, Sgarlata, & Francis, 2016). At present, the number of dementia patients is approximately 35.6 million globally, and dementia caused by AD accounts for 60%–70%. The main pathological change in AD is the formation of extracellular senile plaques after oligomerization of amyloid β (Aβ) monomer in brain tissue and neurofibrillary tangles caused by excessive phosphorylation of Tau protein in neurons (Crunkhorn, (2017)). It is currently conceived that the pathogenesis of AD is mainly caused by the imbalance between Aβ production and elimination. However, the pathogenesis of AD is still not very clear. Recent studies have found that neuroinflammation plays an important role in the development of AD, which has become a novel direction of AD research and treatment (Eikelenboom et al., 2002; Zhang & Jiang, 2015).

The glial cells of central nervous system (CNS) mainly consist of astrocytes (AS), oligodendrocytes (OD), and microglia (MG) (Bordone et al., 2017). MG is a type of barrier cells of CNS. MG is generally in a resting state and acts as an antigen‐presenting cell to participate in the inflammatory response by secreting cytokines and signals in the case of brain tissue damage and infection (Heuss et al., 2018). It has been validated that a large amount of MG is accumulated around the Aβ plaque in AD, and MG is involved in clearing Aβ. MG can clear Aβ by LDL receptor (LRPs)‐mediated signaling; however, in the case of AD progression and further aggregation of Aβ, the recognition ability and phagocytic ability of MG are weakened. At this point, the massive activation of MG can form a large number of inflammatory factors, including interleukins, such as interleukin‐1β (IL‐1β), interleukin‐18 (IL‐18), interleukin‐6 (IL‐6), and tumor necrosis factor‐α (TNF‐α) (Keren‐Shaul et al., 2017; Sarlus & Heneka, 2017). It has also been found that the removal of MG from AD patient, the deposition of Aβ, is not significantly relieved, while neuronal dendritic spine loss is improved, neuroinflammation is alleviated, and memory function impairment is attenuated (Prokop, Miller, & Heppner, 2013), indicating that MG could be regulated as the source of neuroinflammation. In the inflammatory response, pyroptosis is a novel inflammatory death pattern, which mainly depends on the caspase family (Man, Karki, & Thirumalaâ‐Devi, 2017). Among them, caspase‐1 can mediate the cleavage of downstream gasdermin D (GSDMD) and pro‐IL‐1β, and cleaved GSDMD forms p30‐GSDMD, which forms oligomers by oligomerization, anchors on the cell membrane, leading to the formation of cell membrane pore, increasing cellular osmotic pressure, membrane rupture, and release of a large amount of inflammation factors (Aglietti et al., 2016). Among them, Nod‐like receptors (NLRs) are a type of plasma membrane recognition receptor, and the formed inflammasome can recruit ASC and pro‐caspase‐1 to form and cleave mature caspase‐1 protein. Therefore, NLRPs inflammasome have an important role in the general pyroptosis process (Naji et al., 2016; Wree et al., 2014). NLRP3 inflammasome‐mediated inflammatory pyroptosis exists in MGs, which is the most critical regulatory pattern of the release of neuroinflammatory factors.

MicroRNAs, noncoding RNAs with 18–22 nt in length, mainly recognize the 3'UTR sequence of mRNA of target genes to inhibit the transcription and translation process of mRNA, thereby regulating the production of target protein. At present, miRNAs have been found to play important roles in various diseases, and the abnormal expression of a large number of miRNAs has also been found in AD studies (Chang, Wang, & Zhu, 2017); however, the regulation mechanism of miRNAs in AD‐related neuroinflammation has rarely been reported. Our team has previously found the low expression of miRNA‐22 in peripheral blood of AD patients, and the expression of inflammatory factors is negatively correlated with miRNA‐22. Therefore, it is cautiously speculated that miRNA‐22 may play a certain role in the regulation of inflammatory response. Thus, in this study, we explored the regulatory roles and modes of miRNA‐22 in AD‐related neuroinflammation.

## 2 MATERIALS AND METHODS

### 2.1 Detection of serum miRNA‐22 and inflammatory factors in AD patients

A total of 33 AD patients were collected between January 2017 and December 2018 (age: 58–88 years old, 69.87 ± 7.85). Patients were screened using the Mini‐Mental State Scale (MMSE), who met the diagnostic criteria of DSM‐IV dementia and AD in NINCDS‐ADRDA after excluding vascular dementia, glioma, other neurological diseases, and other system‐induced cognitive impairment. AD patients with cognitive impairment were evaluated by the Montreal Cognitive Assessment Scale (MoCA), where MoCA score <26 indicated cognitive impairment. Meanwhile, 30 healthy people were selected as controls (age: 55–86 years old, 65.35 ± 6.93). AD patients and control population were fasted for 8 hr before extraction of peripheral blood (5 ml of venous blood). After incubation for 30 min, the blood sample was centrifuged at 3,000 *g* for 10 min and centrifuged at 16,000 *g* for 10 min at 4°C to obtain serum, which was further stored at −80°C. The total serum RNA was extracted using the miRNeasy serum RNA extraction kit (Qiagen), followed by determination of purification and concentration of RNA sample using a NanoDrop Lite Spectrophotometer (Thermo). The cDNA reverse transcription assay was performed using the miScript II RT Kit (Qiagen). In brief, the reaction system was 20 μl, consisting of 4 μl of 5xmiScript Hispes solution, 2 μl of 10xmiScript Nucleies mixture, 2 μl of miScript reverse transcription mixture, 2 μl of RNase‐free water, and 10 μl of template RNA. The reverse transcription was performed at 37°C for 60 min and subsequently at 95°C for 5 min. RT‐PCR of miRNA‐122 was performed using miScript SYBR Green PCR Kit (Qiagen). The reaction conditions were 95°C for 15 min; 94°C for 15 s; 55°C for 30 s; and 70°C for 30 s, 40 cycles. The primer sequence of miRNA‐22 was shown in the following: F: 5′‐AAGCTGCCAGTTGAAGAACTGTA‐3′; R: 5′‐GCTGTCAACGATACGCTACGTAAC‐3′. The primer sequence of the internal control U6 was shown as follows: F: 5′‐CGCTTCGGCAGCACATATA‐3′; R: 5′‐TTCACGAATTTGCGTGTCAT‐3′. The relative expression of miRNA‐22 was calculated using the 2−∆∆Ct method, ∆Ct = −Ct(miRNA‐22) − Ct(U6), ∆∆Ct = (Ct of target gene − Ct of internal control) experimental group − (Ct of target gene − Ct internal control) control.

The serum inflammatory factors of patients were determined by ELISA. The levels of IL‐1β, IL‐18, and TNF‐α were detected by ELISA kit (Nanjing Jian Biotechnology Co., Ltd.) according to the manufacturers’ instruction. The absorbance was measured at 450 nm using microplate reader (BioTek), and results of inflammatory factor levels were shown as pg/ml.

The study was approved by Ethics Committee.

### 2.2 Expression changes in miRNA‐22 and GSDMD in APP/PS1 double transgenic mouse model

APP/PS1 is a commonly used mouse model among AD models. The hippocampus of mouse was resected from APP/PS1 mice at 3, 6, and 9 weeks old, followed by detection of the expression of miRNA‐22 and GSDMD by RT‐PCR. The primer sequence of miRNA‐22 and the internal control U6 primer sequence were as described above. The primer sequence of GSDMD was shown as follows: F: 5′‐TGTCGTCGATGGGAACATTCAG‐3′, R: 5′‐ATTCATGGAGGCACTGGAACTGTC‐3′.

### 2.3 Effect of miRNA‐22 on pyroptosis and inflammatory factor release in MGs

Mouse primary microglia MGs (Punuosai Life Technology Co., Ltd.) were used for the assay. Briefly, MG cells were transfected with miRNA‐22 mimic and miRNA‐22 inhibitor (GenePharm Co., Ltd.). In terms of pyroptosis induction, MGs were pretreated with 1 μg/ml of lipopolysaccharide (LPS) (Sigma) for 5 hr, followed by addition of 10 μM of Nigericin (MCE).

1. Lactate dehydrogenase cytotoxicity assay: LDH kit (Solarbio) was used to detect cytotoxicity. The LDH release rate was detected 2 hr after the addition of Nigericin for pyroptosis induction.
2. Propidium iodide (PI) absorption rate assay: The cell permeability of pyroptotic cells was increased, and PI can penetrate into the cell membrane pore for staining; therefore, the cell permeability can be detected by the relative absorption rate of PI. Within 2 hr after Nigericin intervention, PI absorption rate was detected every 10 min (a total of 12 time points). In brief, 1 μg/ml of PI, 120 nM of NaCl, 5 mM of glucose, 1.5 mM of CaCl_2_, 1 mM of magnesium chloride, and 0.1% bovine serum albumin (BSA) were used for this assay. Absorbance was determined at 533/617 nm. PI absorption rate (%) = (OD_Sample_ − OD_background_)/(OD_maximum_ − OD_background_).
3. Immunofluorescence (IF) staining of GSDMD: IF staining of cells was performed, after placing the coverslips in 6‐well plates. After LPS pretreatment for 5 hr, 10 μM of Nigericin was added for further intervention for 2 hr. Cells were fixed with freshly prepared 4% paraformaldehyde (PFA) for 10 min, washed with PBS for three times, permeabilized with 0.2% Triton X‐100 for 10 min, blocked with 2% BSA for 30 min, incubated with anti‐GSDMD monoclonal antibody (dilution 1:300, Abcam) at room temperature for 1 hr, washed with PBS for three times, labeled with IgG antibody (Abcam), stained with 0.5 μg/ml DAPI staining reagent (Solarbio), washed with PBS for two times, sealed, and observed under microscope. Since p30‐GSDMD was p30 protein after cleavage, it can be labeled with anti‐GSDMD monoclonal antibody. p30‐GSDMD was mainly localized in the cell membrane, which was also distributed in the cytoplasm.
4. Detection of inflammatory factor expression from culture medium by ELISA: Cells were treated with Nigericin for 2 hr, and the medium was sampled at 0.5 min for centrifugation at 3,000 *g*. Afterward, the supernatant was collected to detect the levels of inflammatory factors, including IL‐1β, IL‐18, and TNF‐α by ELISA kit (Nanjing Jiancheng Biotechnology Co., Ltd.) according to the manufacturers’ instruction. The absorbance was measured at 450 nm using a microplate reader (BioTek), and the result was shown as pg/ml.
5. Detection of pyroptotic level by PI and Hoechst 33258 staining: After pyroptosis induction by LPS and Nigericin in MG cells, cells were stained after intervention with Nigericin for 2 hr. After discarding medium, cells were washed with PBS for two times, stained with Hoechst 33258 staining solution (dilution 1:100, Beyotime Biotechnology Co., Ltd.) for 15 min, and washed with PBS for two times, followed by observation under microscope, which should display as blue fluorescence for positive cells. For PI staining, 1 μg/ml of PI staining reagent (Beyotime Biotechnology Co., Ltd.) was used for staining for 30 min, followed by washing with PBS for two times and observation, which should display as red fluorescence for positive cells. The above two staining methods were used to detect the number of pyroptotic cells, and the final results were shown as the number of positive cells under fluorescence microscope.
6. Detection of the relative protein expression by Western blot: Pyroptosis was induced according to the above protocol. After Nigericin intervention for 2 hr, cells were collected and washed twice with PBS, lysed with 1.0 ml of precooled RIPA lysate (Beyotime Biotechnology Co., Ltd.) on ice for 30 min, and centrifuged at 10,000 *g* for 15 min, followed by quantification of the supernatant by BCA kit (Beyotime Biotechnology Co., Ltd.). Protein sample was mixed with 5x loading buffer (20 μl in total), boiled for 8 min, subjected to SDS‐PAGE at 80 V and then 120 V, and transferred to PVDF membrane at 300 mA for 0.5‐2 hr. The PVDF membranes were blocked with 5% skim milk for 2 hr and incubated with primary antibodies diluted in TBST. Afterward, the membranes were washed with TBST for two times and incubated with horseradish peroxidase (HRP)‐labeled goat anti‐rabbit secondary antibody (Abcam). Afterward, ECL method was used, followed by analysis of the optical density using Image Pro‐Plus 6.0 software. GAPDH was used as the internal control. The results were shown as comparison of optical density values between the target protein and the internal control protein. The primary antibodies included anti‐GSDMD and anti‐p30‐GSDMD monoclonal antibodies (dilution 1:500, Abcam); anti‐NLRP3 and anti‐caspase‐1 monoclonal antibodies (dilution 1:800, Abcam); and anti‐IL‐1β, anti‐IL‐18, and anti‐TNF‐α monoclonal antibodies (dilution 1:500, Abcam). The secondary antibody, HRP‐labeled IgG antibody, was diluted at 1:1,000 [Abcam]).

### 2.4 Effects of silencing GSDMD by siRNA on pyroptosis in MG cells

To investigate the effects of GSDMD on pyroptosis and inflammatory factor release in MG cells, siRNA (GenePharm Co., Ltd.) was used to silence GSDMD expression. The verified siRNA sequence with relatively good silencing efficacy was as follows: CTCCATGAATGTGTGTATACT. MG cells were seeded into 24‐well plates at confluency of 30%. The total medium before transfection was 0.45 ml. A 50 pmol (0.67 μg) of siRNA was added to Opti‐MEM (Sigma) at a final volume of 25 μl. A 1 μl of Entranster‐R4000 was added to 24 μl of Opti‐MEM, followed by incubation at room temperature for 5 min. The diluted siRNA and the diluted Entranster‐R4000 dilution were mixed for incubation at room temperature for 15 min. A 50 μl of the transfection solution was added to 0.45 ml of complete medium to mix well. After transfection for 6 hr, the MG cell state was good. MG cells were incubated for 48 hr, followed by detection of the expression level of GSDMD mRNA.

### 2.5 Detection of the binding relationship between miRNA‐22 and GSDMD by luciferase reporter gene assay

psiCHECK‐2 luciferase reporter vector and pMD18‐T vector harboring the 3′ UTR sequence of target gene were double digested by Not I and Xho I. To be specific, the digestion system consisted of 1 μl of Not I and 1 μl of Xho I, 2 μl of 10 × H Buffer and 2 μl of 0.1% BSA, DNA <1 μg, 2 μl of 0.1% Triton X‐100, and ddH_2_O up to 20 μl. After digestion at 37°C for 2 hr, the reaction was stopped by adding 2 μl of 10x loading buffer. The product was subjected to 1% agarose gel electrophoresis, followed by collection and purification of the luciferase vector and the target sequence fragment for subsequent ligation reaction. PsiCHECK‐2 luciferase reporter gene was ligated to the 3′UTR of GSDMD mRNA. The ligation reaction system consisted of 2.5 μl of 10x T4 DNA ligase buffer, 0.3 pmol of DNA fragment, 0.3 pmol of vector DNA, 1 μl of T4 DNA Ligase, and ddH_2_O up to 20 μl at 16°C water bath overnight. The ligation product was transformed into competent cells, followed by plasmid extraction for identification of Not I and Xho I.

The constructed GSDMD luciferase reporter vector was used as a template to perform mutation of the miRNA‐22 targeting binding sequence on the 3′UTR.

Luciferase reporter vector and miRNA‐22 transfection: HEK‐293T cells (Punuosai Biotechnology Co., Ltd.) were inoculated into 24‐well plates. 0.2 μg of GSDMD luciferase reporter vector and mutant reporter vector, 0.8 μg of miRNA‐22, and controls were diluted in 50 μl serum‐free antibiotics‐free medium. Lipofectamine 2000 was diluted in 50 μl serum‐free antibiotics‐free medium for incubation at room temperature for 5 min. The plasmid and the diluted Lipofectamine 2000 were mixed and incubated at room temperature for 30 min. HEK‐293T cells were cultured in DMEM medium, and the mixture of vector and liposome was added to cells. After transfection for 6 hr, the medium was changed for further incubation.

Detection of luciferase activity: 5x passive lysis buffer (PLB) was diluted to 1x. Luciferase assay substrate was dissolved in 10 ml of luciferase assay buffer II and mixed well. 50x Stop & Glo substrate was diluted to 1x using Stop & Glo Buffer. After cotransfection for 48 hr, the medium in the 24‐well plate was discarded and added with the prepared luciferase assay buffer II, followed by detection of luciferase activity after mixing. Afterward, 100 μl of Stop & Glo Buffer was added and mixed well to detect Renilla luciferase activity.

### 2.6 GSDMD rescue assay validated the regulatory relationship between miRNA‐22 and GSDMD

miRNA‐22 mimic transfection could reduce the expression of GSDMD. GV362 plasmid vector (for GSDMD overexpression) and mimic were cotransfected into MG cells to validate the resistance of miRNA‐22 after GSDMD overexpression.

Amplification and extraction of plasmid: A 100 μl of *E. coli* (Jikai Gene Chemical Technology Co., Ltd.) was added to 10 ml of LB medium for shaking for 24 hr. The bacterial solution was collected to extract plasmid. In brief, the bacterial solution was centrifuged at 4°C for 15 min, added with 0.3 ml buffer P1 and 0.3 ml buffer P2, and incubated for 5 min after mixing well, followed by washing washed with QIAGEN‐tip. After adding 2.0 ml of buffer QC and 0.8 ml of buffer QF to elute DNA into the RNase/DNase‐free EP tube, 1.0 ml of 70% ethanol was added to wash DNA fragment, centrifuged at 4°C for 10 min, and dried. Plasmid transfection: MG cells were seeded into 6‐well plates. Opti‐MEM medium and HiPerFect transfection reagent were mixed, and 250 μl of Opti‐MEM and 10 μl of GSDMD overexpression plasmid were mixed for incubation for 5 min. The diluted transfection reagent and diluted plasmid were incubated for 20 min. Medium was discarded, and cells were added with the mixed Opti‐MEME for routine incubation for 72 hr. miRNA‐22 mimic was transfected in line with the above procedure.

### 2.7 Effect of miRNA‐22 on memory ability and neuroinflammation in APP/PS1 mice

APP/PS1 double transgenic mice (Jackson) were maintained in JiaXing University Animal Experimental Center. Ten APP/PS1 double transgenic mice of 20‐week age were anesthetized with chloral hydrate, and four mice were injected with miRNA‐22 mimic, while the remaining four were routinely housed. miRNA‐22 mimic was dissolved to the sterile ddH_2_O solution and vortexed for 10 s to form a final concentration of 10 pmol/μl. The mice were fixed, and the stereotaxic map of the mouse brain was used. The left ventricle was located at 0.6 mm posterior bregma, 0.8 mm left to the midline, and 2.4 mm under the skull. A 4 mg/kg of miRNA‐22 mimic was pipetted with a microsyringe and the needle remaining for 1 min. After the mice were awakened, the control mice and the miRNA‐22 mimic‐intervened mice were housed in separate cages.

The Morris water maze test was used to measure the memory ability of mice. Morris water maze and video system (Feidi Biotechnology Co., Ltd.), the water maze consists of a circular pool, platform, and recording system. The pool had a diameter of 120 cm, a height of 40 cm, and a water depth of 30 cm. The inner wall of the pool was black, and the temperature was kept at 20°C. The experiment was performed 1 cm underwater, and the experimental platform was performed at the midpoint of the four quadrants. Mice were subjected to adaptive training one day before the experiment. Each mouse was trained once, which was allowed to swim from the entrance for 60 s. If the mouse could not find the platform, it was artificially guided to stand on the platform for 20 s. If the mouse was able to find the platform, then, after standing on the platform for 20 s, it was placed back cage. For navigation test, which lasted for seven days, each mouse was examined four times a day (two times in the morning and another two times in the afternoon). The platform was placed in the fourth quadrant. The time interval of entrance with finding and boarding the platform was recorded. If the mouse could not find the platform within 60 s, then it was guided to the platform and further stood for 20 s. Escape latency (EL) was defined as the time interval between entrance into the water and finding the platform. In terms of space exploration experiment, the platform was removed after the navigation test, and mouse was allowed to enter the entrance. Afterward, investigators recorded the times of crossing the fourth quadrant and the time of staying on the original platform within 60 s. Mouse behavioral test was performed at 1, 3, 5, and 7 after miRNA‐22 mimic intervention.

After the mice were sacrificed, the hippocampus tissues of mice were quickly resected, and protein was extracted to determine the relative protein expression by Western blot according to the above procedures. Pyroptosis key protein, GSDMD and p30‐GSDMD, NLRP3 inflammasome key protein, NLRP3 and caspase‐1, inflammatory factors, IL‐1β, IL‐18, and TNF‐α were detected.

### 2.8 Statistical analysis

SPSS 21.0 software was used for statistical analysis. Chi‐squared test and Fisher exact probability method were used to analyze clinicopathological data. Measurement data were expressed as mean ± standard deviation ( $\bar{x}$ ± s). One‐way ANOVA was used for comparison among multiple groups. SNK test was used for pairwise comparison. A *p* < .05 was considered as statistically significant.

## 3 RESULTS

### 3.1 Expression and correlation of circulating miRNA‐22 and inflammatory factors in AD patients

The level of circulating miRNA‐22 in AD patients was 0.264 ± 0.09, which was significantly down‐regulated compared to that of healthy population (0.470 ± 0.11) (*p* < .001). The expression levels of inflammatory factors, IL‐18, IL‐1β, and TNF‐α in AD patients, were 17.235 ± 3.12 pg/ml, 10.541 ± 2.15 pg/ml, and 21.445 ± 3.65 pg/ml, respectively, which was significantly up‐regulated compared with healthy population (the expression levels of IL‐18, IL‐1β, and TNF‐α were 8.766 ± 1.87 pg/ml, 4.394 ± 0.89 pg/ml, and 12.022 ± 1.87 pg/ml, respectively) (*p* < .001, *p* < .001, and *p* < .001, respectively). Therefore, our data showed that the expression level of circulating miRNA‐22 was significantly down‐regulated in AD patients, while the level of inflammatory factors was significantly up‐regulated in AD patients, compared to those in healthy population.

**FIGURE 1:**
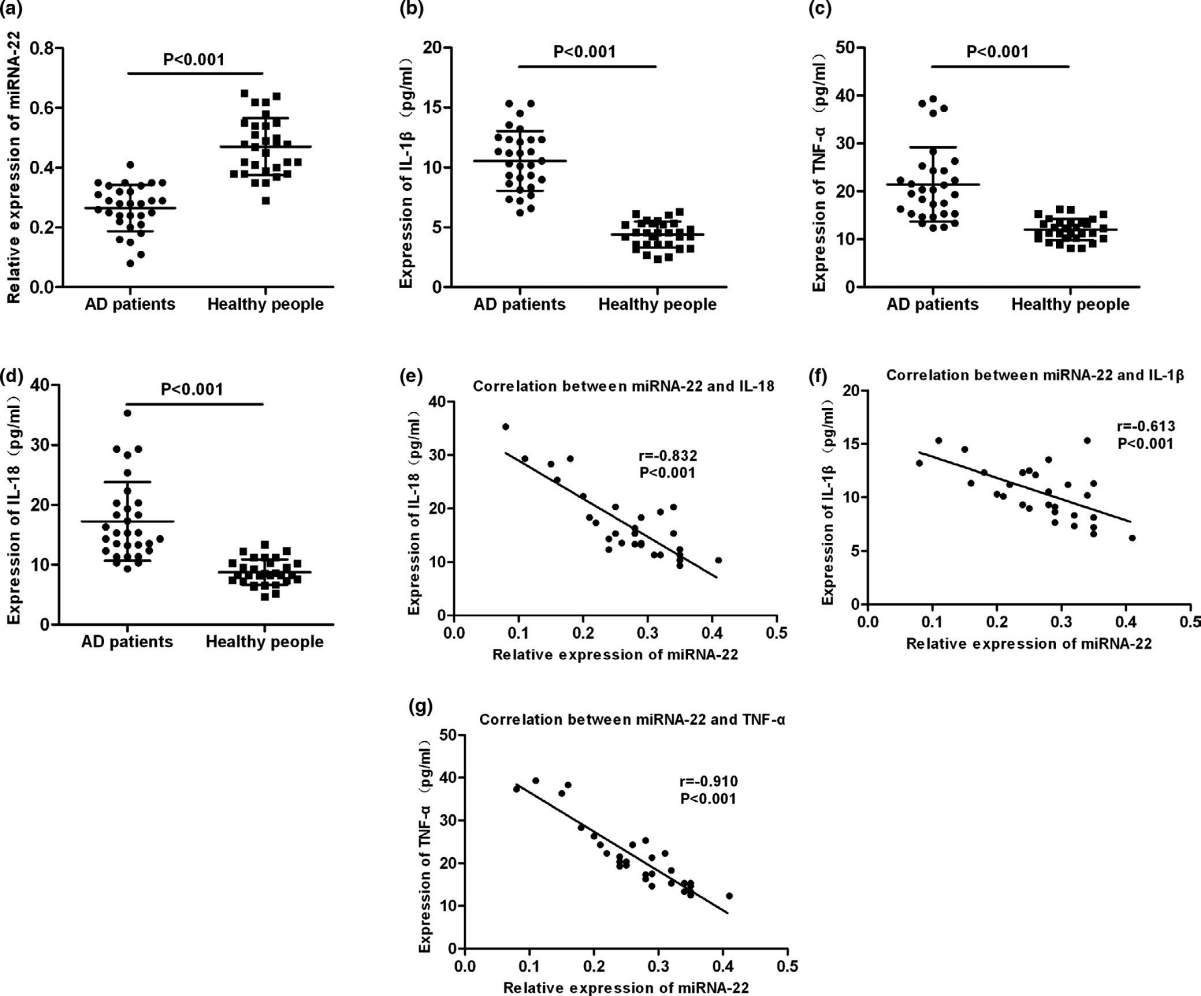
Results of expression and correlation analysis of circulating miRNA‐22 and inflammatory factors. (a) Comparison of relative expression levels of circulating miRNA‐22 between AD patients and healthy people, *p* < .001. (b–d) Comparison of the expression levels of IL‐18, IL‐1β, and TNF‐α between AD patients and healthy people. The expression levels of IL‐18, IL‐1β, and TNF‐α in the peripheral blood were significantly higher in AD patients than those in healthy people, *p* < .001. (e–g) The correlation between circulating miRNA‐22 and the expression of inflammatory factors IL‐18, IL‐1β, and TNF‐α in AD patients. miRNA‐22 was negatively correlated with the expression of IL‐18, IL‐1β, and TNF‐α, *r* = −.832, −.613, and −.910, respectively; *p* < .001, *p* < .001, and *p* < .001, respectively. AD, Alzheimer's disease; TNF‐α, tumor necrosis factor‐α

Correlation analysis between miRNA‐22 and inflammatory factors revealed that miRNA‐22 was negatively correlated with the expression of IL‐18, IL‐1β, and TNF‐α in peripheral blood of patients (*r* = −.832, −.613, and −.910, respectively; *p* < .001, *p* < .001, and *p* < .001, respectively) (shown in Figure 1).

### 3.2 Effects of miRNA‐22 on pyroptosis and release of inflammatory factors in MG cells

**FIGURE 2:**
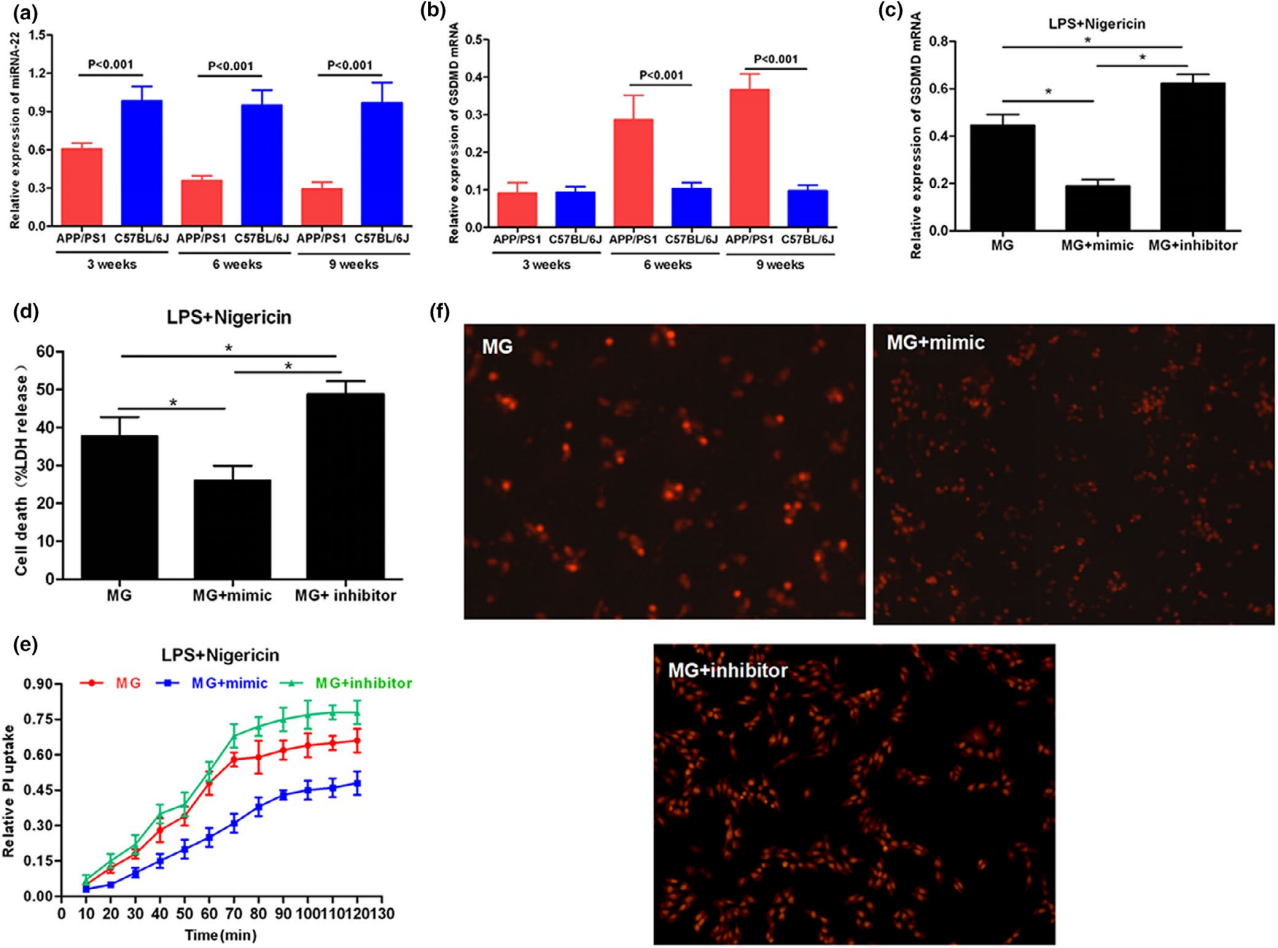
Effect of miRNA‐22 mimic/inhibitor transfection on cell permeability and cytotoxicity. (a) Dynamic expression of miRNA‐22 in hippocampus of APP/PS1 mice (*n* = 3). The expression level of miRNA‐22 in hippocampus was down‐regulated with the increasing age, while the miRNA‐22 expression remained high in normal mice, which was significantly higher than AD mice. Comparison between groups at the same time point, *p* < .05. (b) Dynamic mRNA expression of GSDMD in hippocampus of APP/PS1 mice (*n* = 3). The mRNA expression of GSDMD in hippocampus was up‐regulated with the increasing age, while the mRNA expression of GSDMD remained low in normal mice. Comparison between groups at the same time point, *p* < .05. (c) The expression level of GSDMD mRNA was changed after transfection with mimic/inhibitor (*n* = 3). The mRNA level of GSDMD was down‐regulated after transfection with mimic, while was up‐regulated after transfection with inhibitor. Comparison between groups, *p* < .05. (d) Cytotoxicity detection by LDH release rate assay (*n* = 3). Mimic transfection could significantly down‐regulate cell death rate, and inhibitor transfection could up‐regulate cell death rate, indicating that miRNA‐22 was associated with cytotoxicity in pyroptosis. Comparison between groups, *p* < .05. (e) PI uptake rate assay (*n* = 3). PI absorption was down‐regulated after mimic transfection. (f) PI staining (*n* = 3). The number of positive cells was decreased after mimic transfection, indicating that the PI uptake was down‐regulated. The number of PI positive cells was up‐regulated after inhibitor transfection. AD, Alzheimer's disease; PI, propidium iodide

**FIGURE 3:**
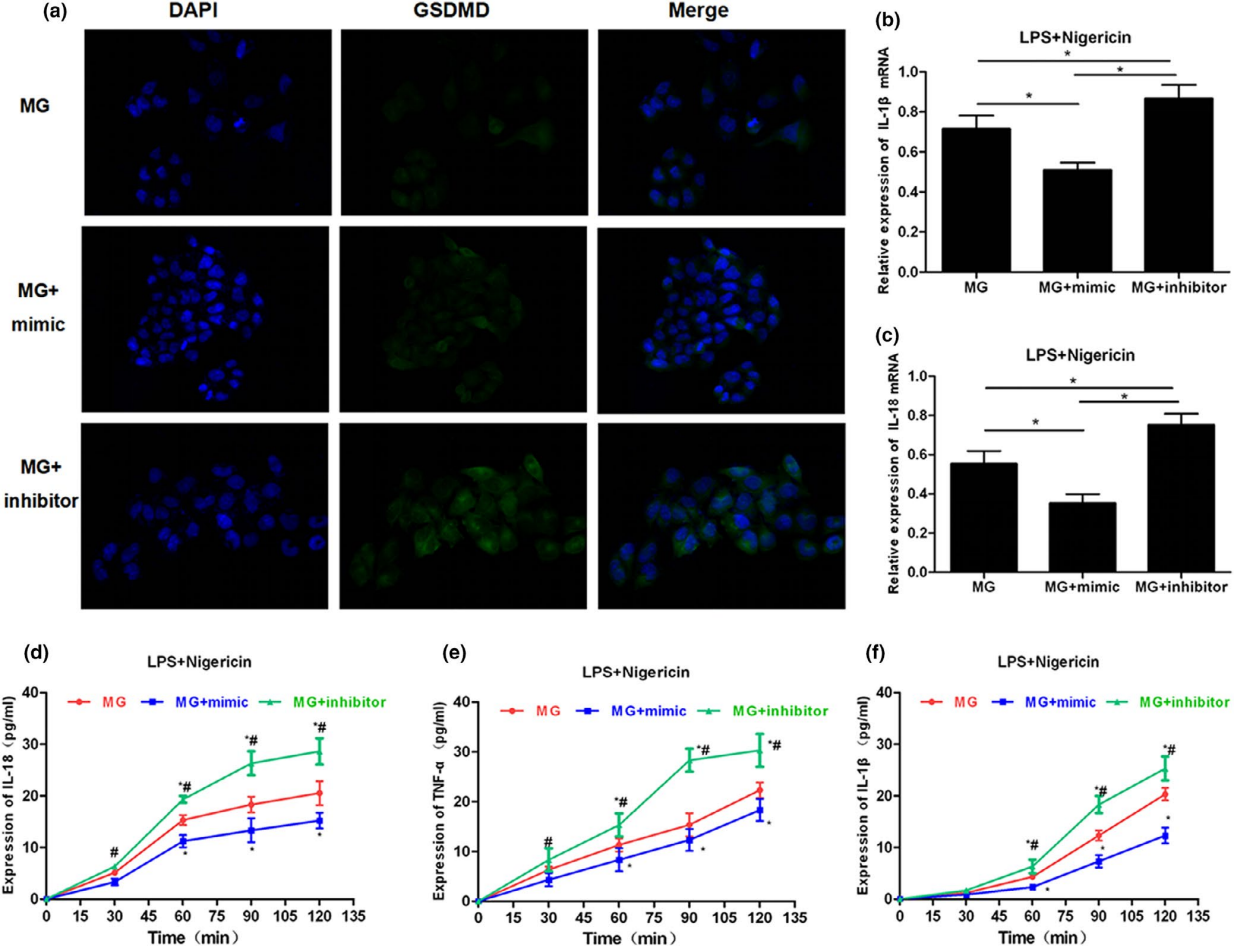
Effects of miRNA‐22 mimic/inhibitor transfection on GSDMD expression and inflammatory factor release. (a) IF staining was used to detect the expression of GSDMD/p30‐GSDMD (*n* = 3). The expression of GSDMD and p30‐GSDMD was down‐regulated after mimic transfection, and the level of p30‐GSDMD was down‐regulated on the membrane surface, while the expression of GSDMD and p30‐GSDMD was up‐regulated in the inhibitor group. (b and c) The mRNA expression of inflammatory cytokines IL‐18 and IL‐1β (*n* = 3). The mRNA level of IL‐18 and IL‐1β was down‐regulated in cells after mimic transfection, which was up‐regulated in inhibitor group. Comparison between groups, *p < .05. (d–f) The dynamic levels of inflammatory factors in the medium (*n* = 3). After mimic transfection, the levels of IL‐18, IL‐1β, and TNF‐α in the medium were significantly down‐regulated, compared to MG and inhibitor groups, and the levels of IL‐18, IL‐1β, and TNF‐α were higher in the inhibitor group than MG group, indicating that miRNA‐22 could inhibit the release of inflammatory factors. Comparison with MG at the same time point, *p < .05; comparison with MG + mimic group, ^#^ *p* < .05. TNF‐α, tumor necrosis factor‐α

**FIGURE 4:**
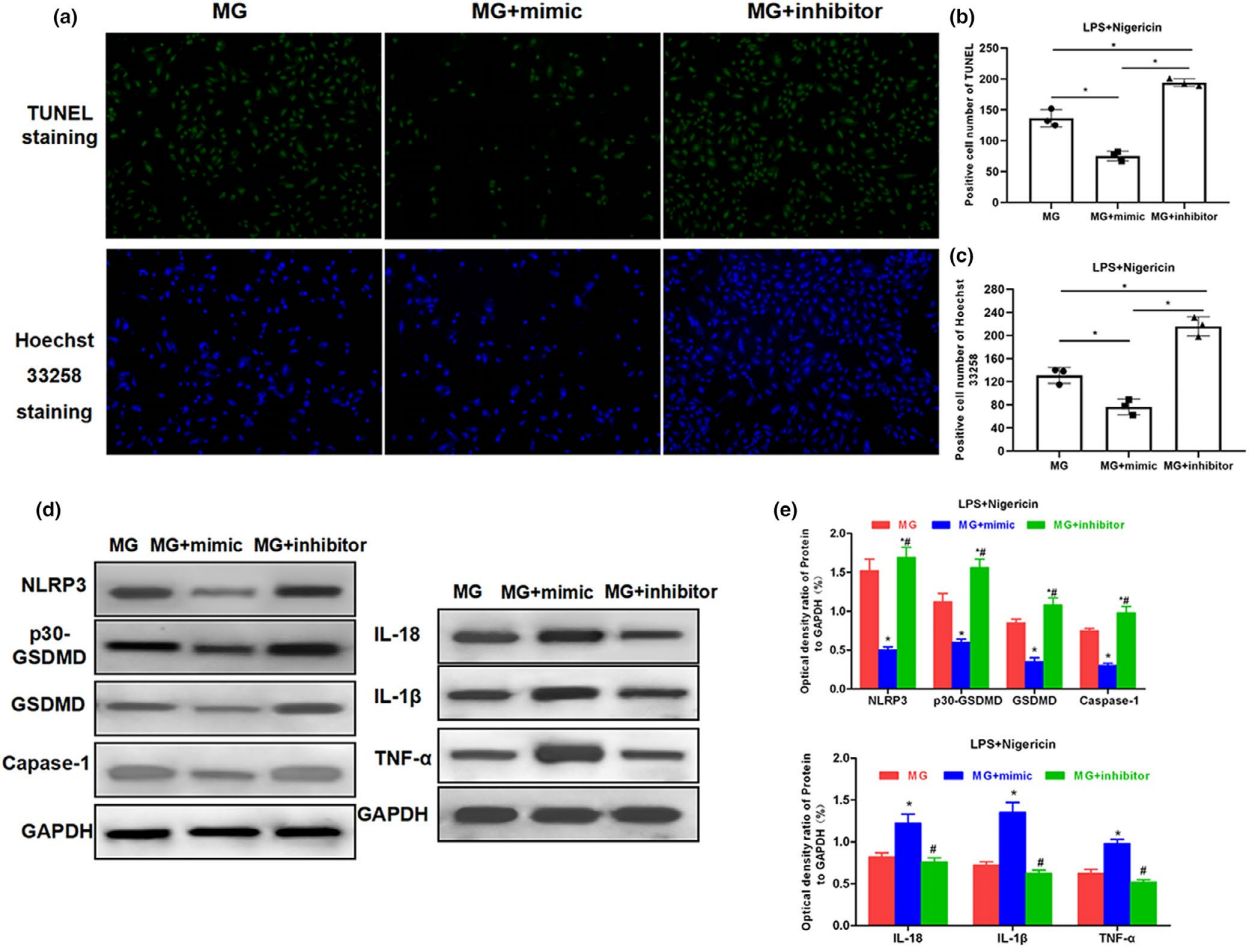
Effect of miRNA‐22 mimic/inhibitor transfection on cell pyroptotic level and the expression of key protein. (a–c) TUNEL staining and Hoechst 33258 staining showed that the number of positive cells was down‐regulated (*n* = 3), and the number of pyroptotic cells was down‐regulated after mimic transfection, while the number of positive cells was up‐regulated after inhibitor transfection. Comparison between groups, *p* < .05. (d and e) Effects on NLRP3 inflammasome, GSDMD protein, and inflammatory factor expression in cells (*n* = 3). Mimic transfection could down‐regulate the expression of NLRP3 and caspase‐1 and simultaneously down‐regulate the expression of GSDMD and p30‐GSDMD. In addition, with the down‐regulated expression of GSDMD and p30‐GSDMD, inflammatory factors were increased in cells, indicating that inflammatory factors were not extracellularly released, reducing the level of inflammatory factors in the medium. Comparison with MG group, *p < .05; comparison with MG + mimic group, ^#^ *p* < .05

APP/PS1 mice were sacrificed by carbon dioxide asphyxiation at 3, 6, and 9 weeks of age, and brain tissue was rapidly separated to resect hippocampus tissues to detect the expression of miRNA‐22 and GSDMD. Normal mice were used as controls. As a result, the relative expression level of miRNA‐22 in the hippocampus of mice was down‐regulated with increasing age, which was significantly different from that of C57BL/6J mice (*p* < .001). At 3 weeks of age, the mRNA level of GSDMD in MP/PS1 mice was not significantly different from that in C57BL/6J mice, while the mRNA levels of GSDMD in MP/PS1 mice were significantly higher than those in C57BL/6J mice at 6 and 9 weeks of age. The above results indicated that the level of miRNA‐22 was down‐regulated, and the mRNA level of GSDMD was up‐regulated with the increasing age of APP/PS1 mice. Mouse MG cells were transfected with miRNA‐22 mimic and miRNA‐22 inhibitor, followed by pyroptosis induction using LPS + Nigericin. After Nigericin induction for 2 hr and mimic transfection, the mRNA expression of GSDMD was down‐regulated in MG cells, and the level of cell damage was down‐regulated (as indicated by LDH release rate). In PI absorption assay, the PI absorption level in mimic group was significantly lower than inhibitor and MG groups with prolonged time, and the PI absorption level in the inhibitor group was significantly higher than MG group, indicating that miRNA‐22 can down‐regulate cell permeability and opening level of the membrane pore, while the inflammatory factor level in the medium was higher in the inhibitor group, but lower in the mimic group, which was positively correlated with the opening of the membrane pore. IF staining also showed that the level of mimic GSDMD/p30‐GSDMD was down‐regulated, while was up‐regulated in the inhibitor group, indicating that miRNA‐22 definitely affected the expression of GSDMD. TUNEL and Hoechst 33258 staining also revealed that mimic could effectively down‐regulate the number of dead cells. In the further detection of key protein expression, mimic could down‐regulate the expression of NLRP3 and caspase‐1, and simultaneously down‐regulate the expression of GSDMD and p30‐GSDMD. With the down‐regulated expression of GSDMD and p30‐GSDMD, the levels of inflammatory factors were increased in cells, which were suggestive that inflammatory factors were not extracellularly released, reducing the level of inflammatory factors in the culture medium. The results were shown in Figures 2, 3, 4.

### 3.3 Effects of GSDMD silencing using siRNA on pyroptosis in MG cells

**FIGURE 5:**
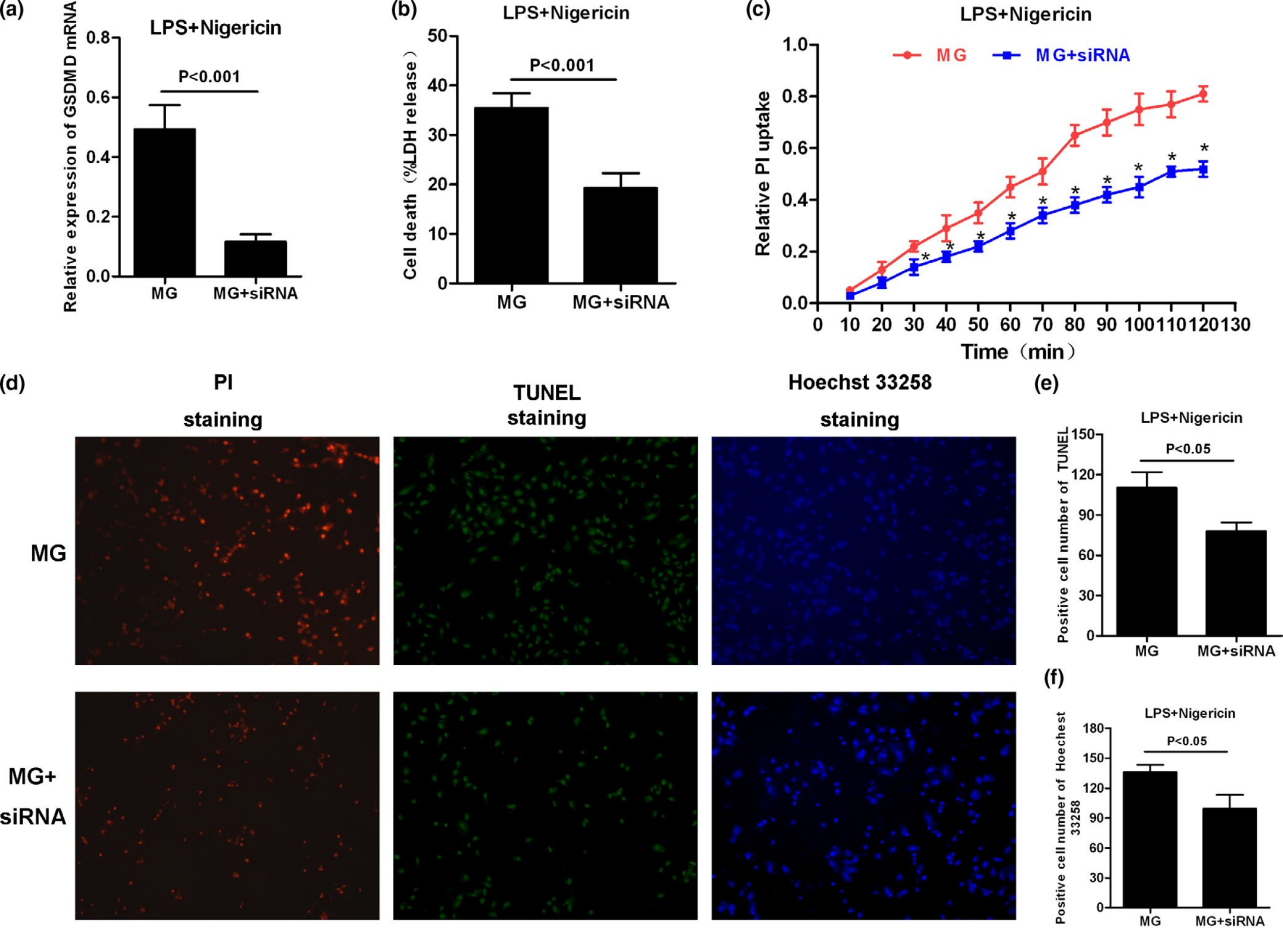
Effects of GSDMD silencing on cell pyroptotic level. (a) Identification of silencing GSDMD efficacy by siRNA (*n* = 3). After siRNA intervention, the mRNA level of GSDMD in MG cells was significantly down‐regulated. Comparison with MG, *p* < .001. (b) Cytotoxicity MG + siRNA was significantly down‐regulated after GSDMD silencing (*n* = 3). Comparison with MG, *p* < .001. (c) PI uptake rate was significantly down‐regulated after GSDMD silencing (*n* = 3). Comparison with MG at the same time point, *p < .05. (d–f): PI uptake and staining (*n* = 3). The number of positive cells stained by TUNEL and Hoechst 33,258 was significantly down‐regulated after GSDMD silencing. Comparison with MG, *p < .05. PI, propidium iodide

**FIGURE 6:**
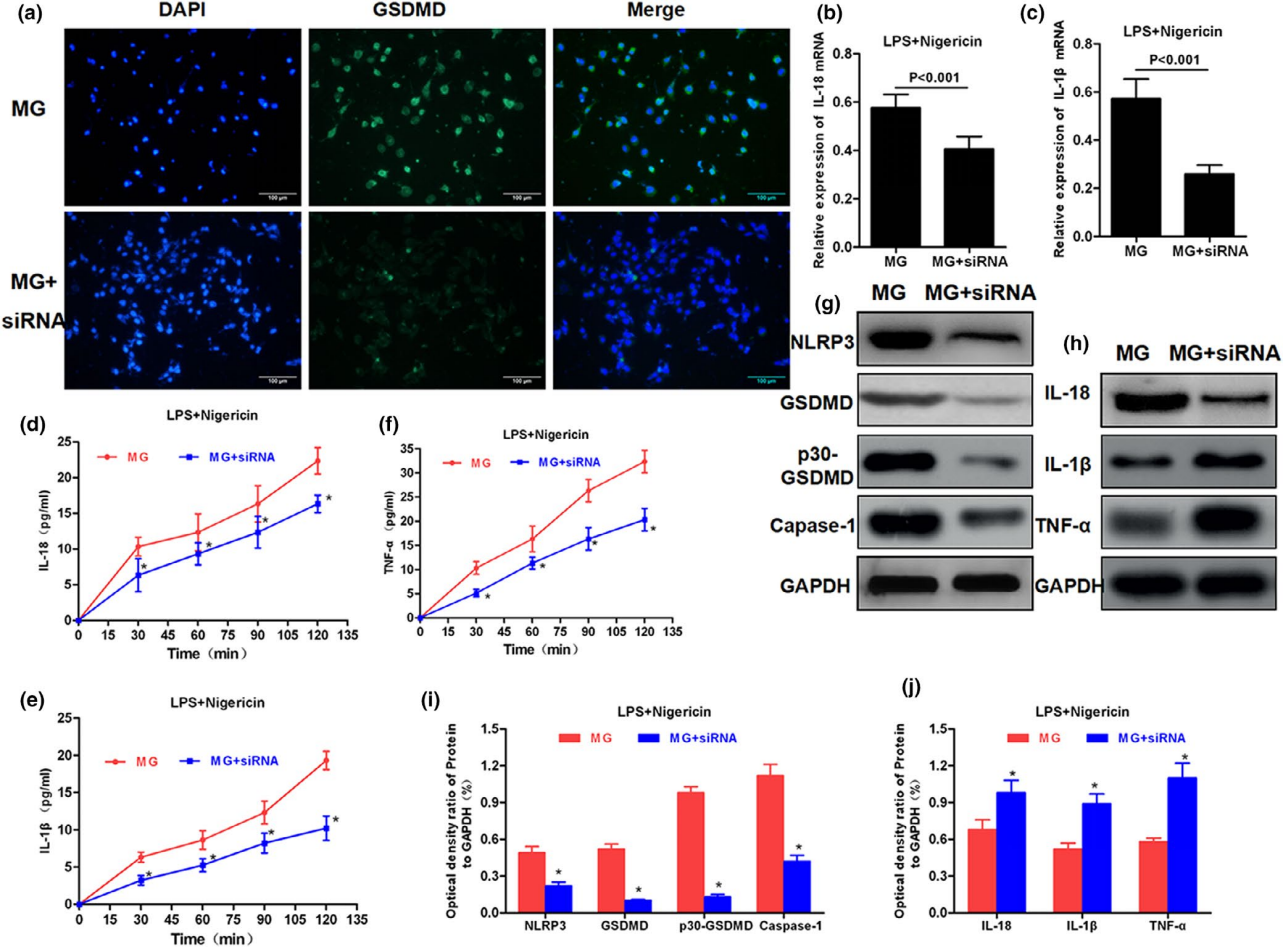
Effects and mechanism of inflammatory factor release after GSDMD silencing. (a) IF staining of GSDMD/p30‐GSDMD (*n* = 3). The expression of GSDMD and p30‐GSDMD was significantly down‐regulated after GSDMD silencing. (b and c) The mRNA expression of inflammatory cytokines IL‐18 and IL‐1β (*n* = 3). The mRNA expression of IL‐18 and IL‐1β was significantly down‐regulated after GSDMD silencing. Comparison with MG, *p* < .001. (d–f) Dynamic change in inflammatory factor expression in the medium (*n* = 3). The levels of IL‐18, IL‐1β, and TNF‐α in the medium were significantly down‐regulated after GSDMD silencing. Comparison with MG at the same time point, *p < .05. (g–j) Effects on NLRP3 inflammasome, GSDMD protein, and inflammatory factors expression (*n* = 3). The expression of NLRP3 and caspase‐1 was down‐regulated after GSDMD silencing, and the expression of GSDMD and p30‐GSDMD was also down‐regulated, while the level of inflammatory factors in cells was increased, indicating that inflammatory factors were not extracellularly released, decreasing the level of inflammatory factors in the medium. Comparison with MG, *p < .05. TNF‐α, tumor necrosis factor‐α

To further investigate the effects of GSDMD on pyroptosis and the release of inflammatory factors, the expression of GSDMD was silenced using siRNA, and pyroptosis was induced by LPS + Nigericin. As a result, after GSDMD silencing, LDH release was down‐regulated and PI uptake was down‐regulated, indicating that the opening level of cellular pore was down‐regulated. TUNEL and Hoechst 33258 staining showed that after GSDMD silencing, the number of positive cells was down‐regulated, suggesting the down‐regulated pyroptotic level in cells. Meanwhile, the levels of inflammatory factors in the medium were down‐regulated, and the levels of key proteins NLRP3, GSDMD, and p30‐GSDMD were down‐regulated, while the levels of intracellular inflammatory factors were higher than those of MG cells, indicating that GSDMD inhibition attenuated the activation of NLRP3 inflammasome, and simultaneously reduced the release of intracellular inflammatory factors and decreased the levels of extracellular inflammatory factors. The results are shown in Figures 5 and 6.

### 3.4 Results of luciferase reporter gene assay and GSDMD rescue assay

**FIGURE 7:**
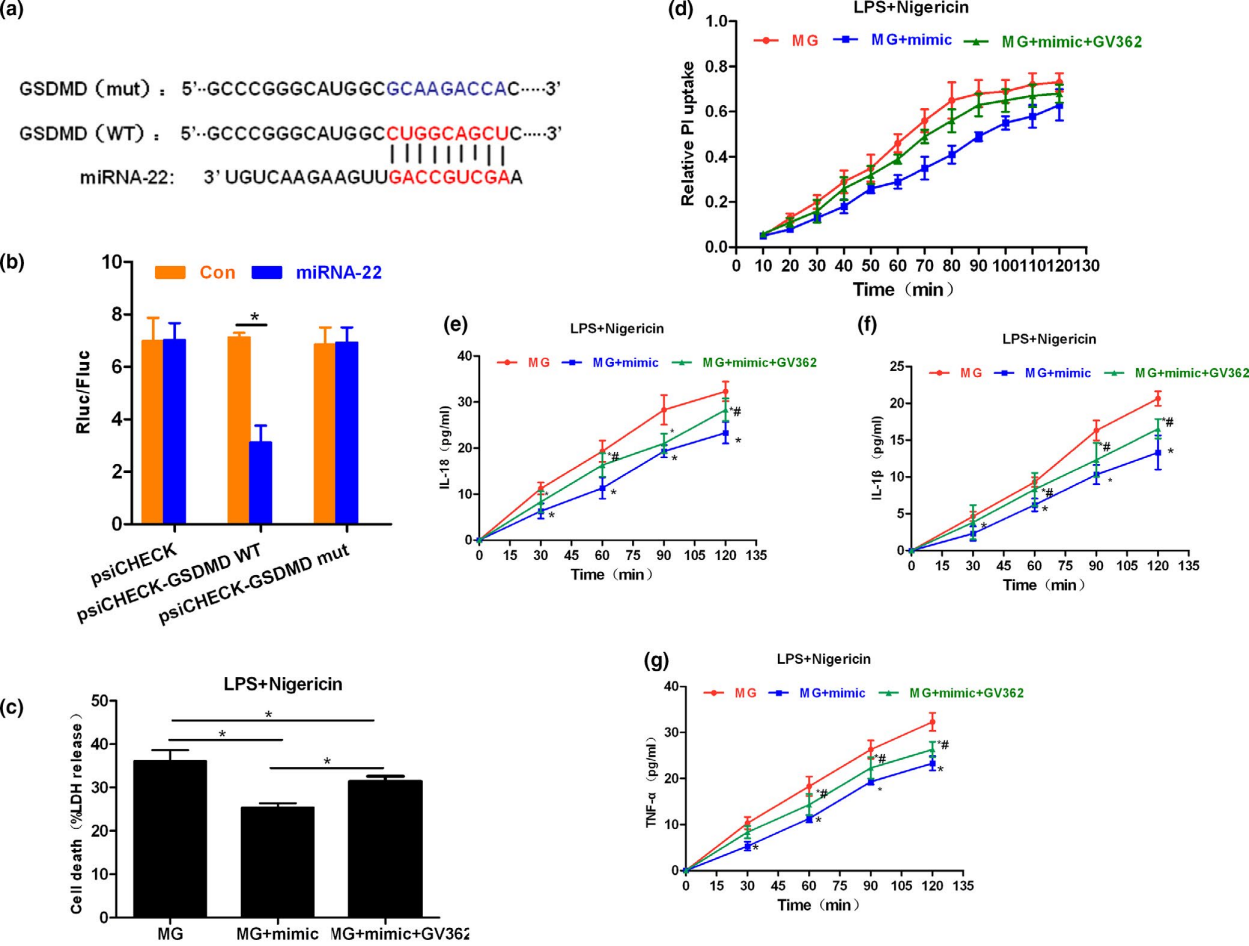
Results of luciferase reporter assay and rescue assay. (a and b) Results of the binding site between miRNA‐22 and GSDMD, mutation site, and luciferase activity assay (*n* = 3). As a result, there was a binding site between miRNA‐22 and GSDMD, and the luciferase activity in Wt was significantly decreased, while there was no significant difference in Mut. Comparison between groups, *p < .05. (c) Cytotoxicity results in the rescue assay (*n* = 3). The overexpression of GSDMD could significantly up‐regulate the cytotoxicity level of pyroptotic cells, which was significantly different between MG + mimic group. Comparison between groups, *p* < .05. (d) PI uptake rate in the rescue assay (*n* = 3). Overexpression of GSDMD could up‐regulate the PI uptake rate of MG cells, indicating that cell membrane pores were open and permeability was improved. Comparison with MG group, *p < .05; comparison with MG + mimic group, #*p* < .05. (e–g) Dynamic changes in inflammatory factors in the medium in the rescue assay (*n* = 3). The overexpression of GSDMD resulted in the significantly down‐regulated levels of inflammatory factors, including IL‐18, IL‐1β, and TNF‐α in the medium. Comparison with MG at the same time point, * *p* < .05; comparison with the MG + mimic group, ^#^ *p* < .05. PI, propidium iodide; TNF‐α, tumor necrosis factor‐α

**FIGURE 8:**
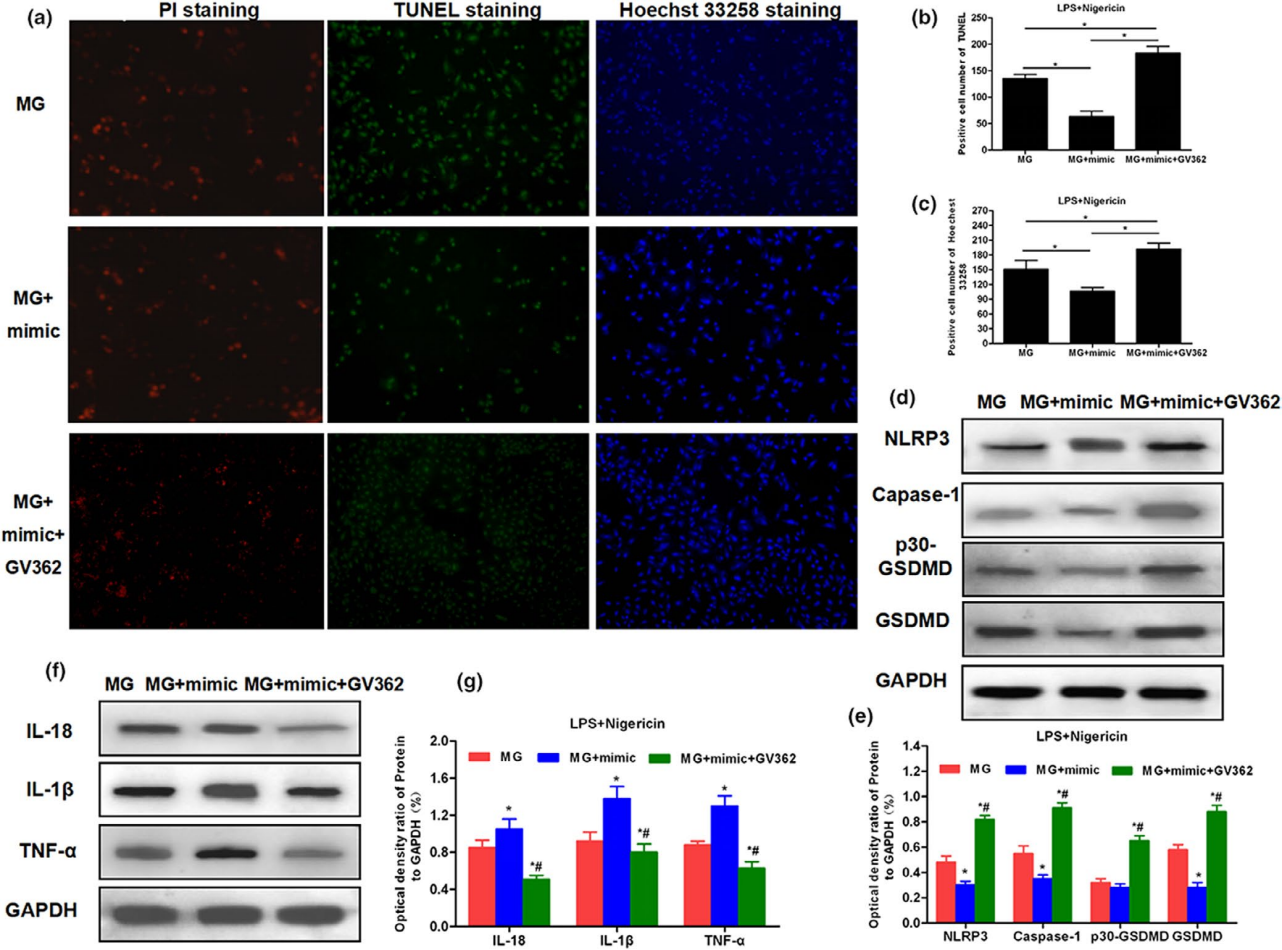
Cell staining and expression of key proteins in the rescue assay. (a–c) Results of PI uptake, TUNEL staining, and Hoechst 33,258 staining (*n* = 3): After overexpression of GSDMD, the PI uptake was increased, and the number of cells with positive TUNEL and Hoechst 33,258 staining was increased. (d–g) The effects on NLRP3 inflammasome, GSDMD protein, and inflammatory factors expression in the rescue assay (*n* = 3). The overexpression of GSDMD caused up‐regulated expression of NLRP3 and caspase‐1, and the up‐regulated expression of GSDMD and p30‐GSDMD, but down‐regulated levels of inflammatory factors in cells, indicating that inflammatory factors were extracellularly released, increasing the level of inflammatory factors in the culture medium. Comparison with MG, *p < .05; comparison with MG + mimic group, ^#^ *p* < .05

Comparison of TargetScan database indicated that miRNA‐22 had a binding site at GSDMD3′, and the binding ability was lost after the mutation of the binding site. The luciferase activity assay also showed that WT had binding ability, while Mut did not, indicating the binding site between miRNA‐22 and GSDMD mRNA. To further investigate whether miRNA‐22 could play a role by inhibiting the expression of GSDMD, GSDMD was overexpressed based on mimic transfection, aiming to rescue the functions of GSDMD. As a result, the overexpression of GSDMD could resist pyroptosis caused by miRNA‐22, which was manifested by increased cytotoxicity in the MG + mimic + GV362 group, increased PI uptake, and up‐regulated expression of inflammatory factors in the medium. Further studies found that overexpression of GSDMD caused the activation of NLRP3 inflammasome and down‐regulated expression of inflammatory factors, indicating that GSDMD overexpression could resist the action of miRNA‐22, further promoting pyroptosis. The results are shown in Figures 7 and 8.

### 3.5 Effects of miRNA‐22 on memory ability and expression of GSDMD‐associated inflammatory factors in APP/PS1 mice

**FIGURE 9:**
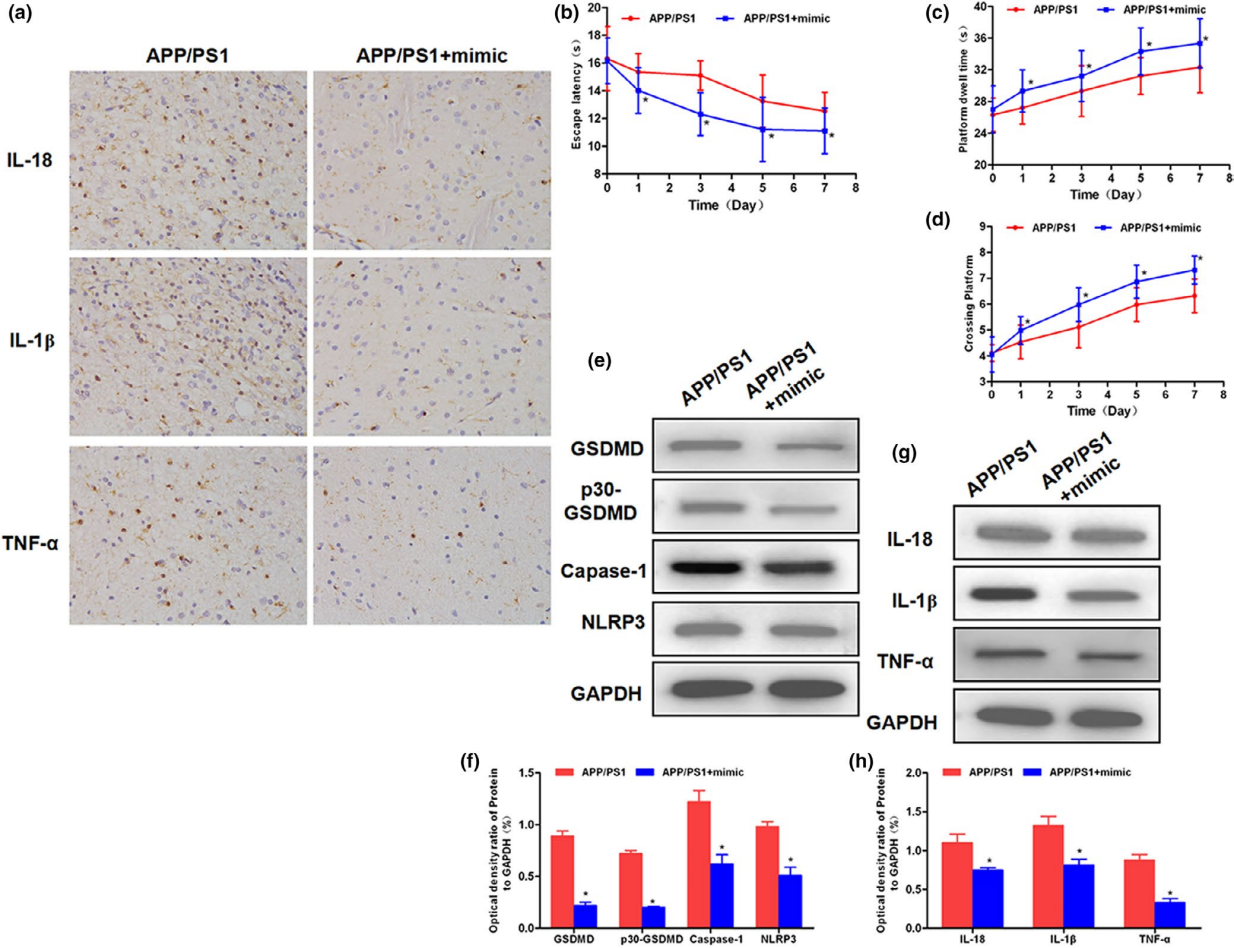
Effect of miRNA‐22 on memory ability and inflammatory factors expression in APP/PS1 mice. (a) IHC staining of hippocampus in mice (*n* = 3): After miRNA‐22 intervention, the levels of inflammatory factors, including IL‐18, IL‐1β, and TNF‐α in the hippocampus of mice, were significantly down‐regulated. (b–d) Morris water maze test in mice (*n* = 5). (b) The escape latency of mice: After mimic intervention, the escape latency was significantly shortened. Comparison with APP/PS1, *p < .05. (c) The retention time on the platform. After the mimic intervention, the retention time on the platform of mice was prolonged. Comparison with APP/PS1, *p < .05. (d) The number of crossing the platform. After mimic intervention, the number of crossing the platform was increased. Comparison with APP/PS1, *p < .05. (e–h) Effects on NLRP3 inflammasome, GSDMD protein, and inflammatory factor expression in mouse hippocampus (*n* = 3). After mimic intervention, the expression of NLRP3 and caspase‐1 was down‐regulated, and the expression of GSDMD and p30‐GSDMD was also down‐regulated, and the expression of inflammatory factors in hippocampus was down‐regulated. Comparison with APP/PS1, *p < .05. PI, propidium iodide; TNF‐α, tumor necrosis factor‐α

The Morris water maze test was a behavioral experiment to detect the memory ability of mice. As a result, after the miRNA‐22 mimic intervention in the lateral ventricle of mice, the memory ability of mice was significantly improved and the EL was significantly shortened, which was significantly down‐regulated with the prolonged intervention time. The retention time and number of crossings the platform of mice were significantly up‐regulated, indicating the improvement of memory ability of mice. The results of IHC and Western blot showed that the expression of inflammatory factors was down‐regulated in mouse hippocampus, and the expression of NLRP3, GSDMD, and p30‐GSDMD was simultaneously down‐regulated, indicating that miRNA‐22 mimic attenuated the activation of NLRP3 inflammasome and the expression of inflammatory factors, thereby inhibiting the overall neuroinflammatory response. The results are shown in Figure 9.

## 4 DISCUSSION

Pyroptosis is a type of inflammatory caspase‐dependent programmed death. In the case of noxious stimulation, intracellular and extracellular signals induce the formation of cytoplasmic inflammasome depending on caspase‐1 or caspase‐4/5/11 (noncanonical pyroptotic pathway), to further activate caspase‐1, promoting the maturation of inflammatory factors such as IL‐18 and IL‐1β, which is released to extracellular space to expand the range of inflammatory response in a cascade form (Baker et al., 2015; Miao, Rajan, & Aderem, 2011). The GSDM protein family is closely related to pyroptosis. GSDM consists of six members. The active N‐terminal of GSDM is exposed after cleaving by caspase protein through various pathways, and N‐terminal oligomerization can form cellular pores on the surface of cell membrane, causing cell swelling and eventually resulting cell death (Rathkey et al., 2017; Rogers et al., 2017). At present, GSDMD is the most studied and well‐defined execution protein related to pyroptosis in the GSDM family. The role of GSDMD can be mediated by canonical caspase‐1 or by noncanonical caspase‐4/5/11. At present, the expression of inflammatory factors, such as IL‐18 and IL‐1β, has been found in various types of cells of multiple types of nervous systems, and caspase‐1 is activated by neural cells under different conditions, thereby causing pyroptosis (Yu‐Lei, Jian‐Hua, & Yan‐Fen, 2018). The role of pyroptosis has been confirmed in neurodegenerative diseases, which has been reflected in AD, Parkinson's disease, and motor neuron disease (Adamczak et al., 2014). β‐amyloid deposition in AD can cause local inflammatory response (Ya et al., 2018), along with the effects from oxidative stress damage and excitatory damage. However, MG plays a major role in neuroinflammation. MG is a centrally unique macrophage that exerts pro‐inflammatory/anti‐inflammatory effects under different pathophysiological environments (Wang et al., 2018).

MicroRNA, 18–22 nt in length, inhibits target gene expression and protein translation by targeting the 3'‐UTR of mRNA. miRNA‐22 is widely studied. The abnormal expression of miRNA‐22 has been detected in various tumors, which can be used as a marker for tumor prognosis (Li, Li, & Sun, 2017; Qiao et al., 2017). However, there are few studies on miRNA‐22 in neurological diseases. In this study, we first found the low expression of circulating miRNA‐22 in AD patients. Intriguingly, the expression level of miRNA‐22 was negatively correlated with inflammatory factors, including IL‐1β, IL‐18, and TNF‐α. TargetScan database comparison and luciferase reporter gene assay revealed that miRNA‐22 could bind to GSDMD; therefore, we first determined the regulatory relationship between miRNA‐22 and GSDMD. In view of the role of GSDMD in pyroptosis, we first validated that miRNA‐22 mimic transfection could inhibit pyroptosis and release of inflammatory factors in MG. Secondly, GSDMD overexpression could resist the functions of miRNA‐22. Thus, we confirmed the regulation between miRNA‐22 and GSDMD in cellular levels of MG. The most important clinical manifestation of AD is cognitive decline. Neuroinflammation is one of the promoting factors for the occurrence and progression of AD. Pyroptosis inhibition can theoretically inhibit the expression of inflammatory factors, thereby improving neuroinflammation and cognitive ability. Thus, in this study, miRNA‐22 mimic was injected into the APP/PS1 mouse model. Consequently, miRNA‐22 mimic could improve the cognitive ability of AD mice and inhibit the expression of inflammatory factors in hippocampus.

Therefore, through a series of studies, we find that miRNA‐22 exerts a certain role in AD. The low expression of miRNA‐22 is related to the occurrence and development of AD. In addition, the expression of miRNA‐22 shows down‐regulation tendency, while the expression of GSDMD is up‐regulated with the increasing age of APP/PS1 mice, which is consistent with the results of human studies. In the mechanism study, miRNA‐22 has been found to interact with GSDMD, the executing protein of pyroptosis. miRNA‐22 could inhibit pyroptosis by suppressing the expression of GSDMD, and in vivo studies have also shown that miRNA‐22 could improve the memory ability of mice.

To sum up, in this study, we find that miRNA‐22 plays a role in AD‐related neuroinflammation. miRNA‐22 can inhibit pyroptosis and the release of inflammatory factors by regulating GSDMD.

## 5 CONCLUSIONS

In this study, we found that the expression level of miRNA‐22 in peripheral blood was lower in AD patients than that in healthy population. The expression of inflammatory factors was higher in AD patients than that in healthy people. In the study of mechanism, we found that miRNA‐22 targeted GSDND to inhibit pyroptosis and thus the release of inflammatory factors. The regulatory mechanism of GSDMD in AD has not been found before. This study found the regulatory mechanism of miRNA‐22 in neuroinflammation and provided new insights into AD‐related inflammation.

## CONFLICTS OF INTEREST

We have no conflicts of interest to declare.

## AUTHORS' CONTRIBUTIONS

Chenyang Han involved in the design and operation of the experiment. Li Guo and Yi Yang processed the data an involved in operation of animal experiment. Qiaobing Guan and Heping shen collected the clinical samples and detected the inflammatory factors. Yongjia sheng and Qingcai jiao involved in the proposal of the subject, the design of the experimental process, and the whole process guidance.

## Data Availability

The data that support the findings of this study are available from the corresponding author upon reasonable request.
